# Theoretical investigation on the addition reaction of the germylenoid H_2_GeLiCl with acetone

**DOI:** 10.3906/kim-2012-35

**Published:** 2021-08-27

**Authors:** Xiaolin ZHANG, Bingfei YAN, Wenzuo LI

**Affiliations:** 1 School of Chemistry and Chemical Engineering, Yantai University, Yantai China

**Keywords:** H_2_GeLiCl, acetone, addition reaction, spiro-Ge-heterocyclic

## Abstract

In this work, theoretical calculations were performed on the addition reaction of the germylenoid H2GeLiCl with acetone. The DFT M06-2X method was used to optimize the geometries of the whole stationary points on the potential energy surfaces and the QCISD method to calculate the single-point energy. The results reveal that the addition reaction of H_2_GeLiCl with acetone firstly generates an oxagermacyclopropane c-H_2_GeOC(CH_3_)_2_ and then c-H_2_GeOC(CH_3_)_2_ further reacts with acetone along two possible pathways, pathway I and pathway II, in which the 2,4-dioxagermolane is formed at the end of pathway I and 2,5-dioxagermolane is formed at the end of pathway II, respectively. According to the calculated barrier heights, we can deduce that the pathway I is more favorable than pathway II. The computational results suggest that this reaction model can provide new inspiration for the synthesis of heterocyclic germanium compounds.

## 1. Introduction

For recent years, germylene and its derivatives have been well studied as a bioactivator in medicine. Germylenoid is a derivative of germylene. Since Lei and Gaspar [1] firstly pointed out that there may exist an intermediate named germylenoid in the reaction of dichlorodimethyl germane with lithium in the presence of substituted 1,3-dienes in 1991, both theoretical and experimental research on germylenoid have been going on for more than 20 years. In 2000, Ichinohe et al. [2] proved that an active germylenoid intermediate *t* -Bu_3_SiGeCl_2_Na played an important role in the reaction of *t* -Bu_3_SiNa with GeCl_2_· dioxane. In 2006, Tokitoh et al. [3,4] pointed out that an important intermediate in the addition reaction of 1,2,4,5-tetrabromobenzene with dilithiogermane Tbt(Dip)GeLi_2_ was germylenoid Tbt(Dip)GeLiBr. In 2012, Fillipou et al. [5] firstly synthesized the zwitterionic germylidene complexes and they believed a germylenoid was one of the reactants in the reaction. There was no one stable germylenoids being prepared experimentally until Sasamori et al. [6] firstly synthesized a stable germylenoid successfully in 2016. A chlorogermylenoid (Fc*GeCl_2_Li) that includes 2,5-bis(3,5- *di* - *t* -butylphenyl) ferrocenyl (Fc*) group was generated in their groundbreaking work and the specific structure and ambident reactivity of this chlorogermylenoid were investigated. With the development of the experimental works about germylenoids, more attention has been paid to theoretical researches [7–32]. In 1999, Qiu et al. [7] firstly studied the germylenoid H_2_GeLiF by ab initio quantum calculations method. By analyzing structures and the solvent effect in different solvents of the germylenoid H_2_GeLiF, Ma et al. [8] obtained four possible stable equilibrium structures in the gas phase in 2007. In 2006, Li et al. [9] firstly came up with this concept for unsaturated germylenoid H_2_C=GeNaF. In 2008, Tan et al. [10] systematically investigated the geometries and isomerization of the germylenoid HN=GeNaF and discussed the insertion reactions with R–H (R = F, OH, NH_2_, CH_3_), and the study came to a conclusion that the relative reactivity of reactants is as follows: H–F > H–OH > H–NH_2 _> H–CH_3_.The structures and properties of other different germylenoids such as H_2_GeLiF [11,12], H_2_GeFMgF [13], H_2_GeZnCl_2 _[14], HP=GeLiF [15], H_2_GeAlCl_3 _[16], and so on, were investigated using theoretical methods. The different reactions such as insertion, elimination, substitution, and addition reactions [11–32] of germylenoids were also analyzed by theoretical calculations. These works provide a lot of useful information for the correct understanding of the structure, properties and reactivity of germylenoid compounds. However, few studies have been done on the addition reactions of germylenoid. Only the addition reactions of some germylenoid with ethylene [13–16,26–31] and formaldehyde [32] have been reported in the literature. Recently, we have calculated the addition reaction of H_2_GeLiCl with acetone and found that the product is a heterocyclic germanium compound, which provides new inspiration for the synthesis of new germanium-containing compounds. 

## 2. Theoretical methods 

The relevant calculation details have been depicted in previous research [16]. The instrument that caused the calculation to be implemented was Gaussian 09 series of programs [33]. The geometries of the whole stationary points were optimized by using the method of density functional theory (DFT) M06-2X [34,35] and 6-311+G (d,p) [36] basis set (BSI). The vibrational frequency was calculated by the same method in order to confirm the minima or saddle points of structures and obtain zero-point energies (ZPEs). The mechanism of the addition reaction pathways was proved by using the IRC [37] analysis to probe every transition state connected with the corresponding stationary points correctly. The single-point computations were carried out using the QCISD [38,39] method with 6-311++G (d,p) basis set (BSII). The given relative energies in this paper were calculated by QCISD/BSII//M06-2X/BSI level and including the M06-2X/BSI calculated ZPEs (without scale) corrections. The molecular electrostatic potentials (MEPs) for the reactants were also calculated at M06-2X/BSI level.

## 3. Results and discussion

The previous calculations [2,11,12] proved that germylenoid H_2_GeLiCl has three equilibrium structures, and the most stable configuration of them is the *p* -complex. That is to say, the *p* -complex structure of H_2_GeLiCl is obviously the target when we investigated the addition reaction of germylenoid H_2_GeLiCl (R1) and acetone CH_3_COCH_3 _(R2). 

**Scheme 1:**
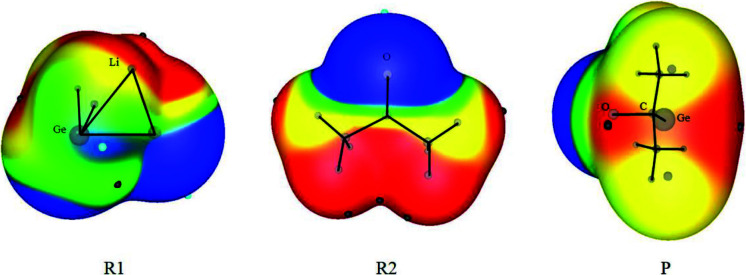
The molecular electrostatic potentials for the reactants (R1, R2, and P) calculated at M06-2X/BSI level (color ranges are: red, greater than 0.03; yellow, between 0.03 and 0; green, between 0 and –0.01; blue, less than 0.01, in eV).

There are two steps in the addition reaction of H_2_GeLiCl with acetone according to the calculation results. In the first step, an oxagermacyclopropane ( *c-* H_2_GeOC(CH_3_)_2_, marked as P) can be generated. In the second step, P can further react with acetone CH_3_COCH_3_ in two possible pathways. The M06-2X/BSI calculated MEPs for R1, R2, and P were shown in Scheme 1. From Scheme 1 it can be seen that there is a positive electrostatic potential region around the Ge atom in R1 and a negative electrostatic potential region around the O atom in R2. The interaction of these two regions will lead to the first step of the reaction of R1 and R2. Interestingly, there are two positive electrostatic potential regions around the Ge atom in P. When the O atoms in acetone approach different positive electrostatic potential regions of P, different reaction pathways (path I and II) will occur. These two steps and two different pathways will be illustrated in detail.

### 3.1. Step 1 of the addition reaction of H2GeLiCl with acetone

The reaction equation for the first step of the reaction of germylenoid H_2_GeLiCl with CH_3_COCH_3_ is depicted as follows: 

H_2_GeLiCl(R1) + CH_3_COCH_3_(R2) → LiCl + *c* -H_2_GeOC(CH_3_)_2_(P)

**Figure 1:**
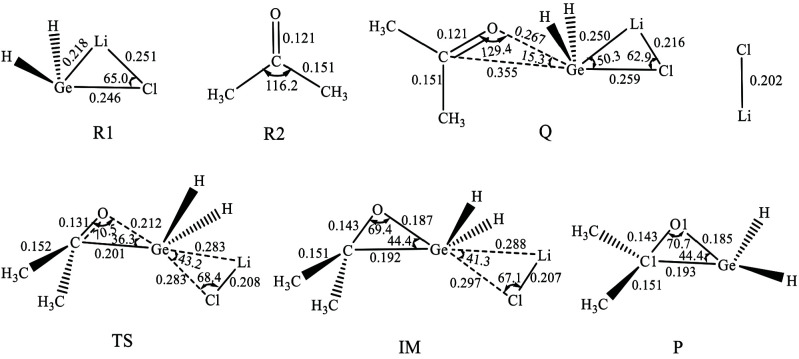
The geometries of the stationary points along the potential energy surfaces of the addition reaction of H_2_GeLiCl and acetone optimized at the M06-2X/6-311 + G (d,p) level (bond lengths are given in nm and angles in degrees).

**Figure 2:**
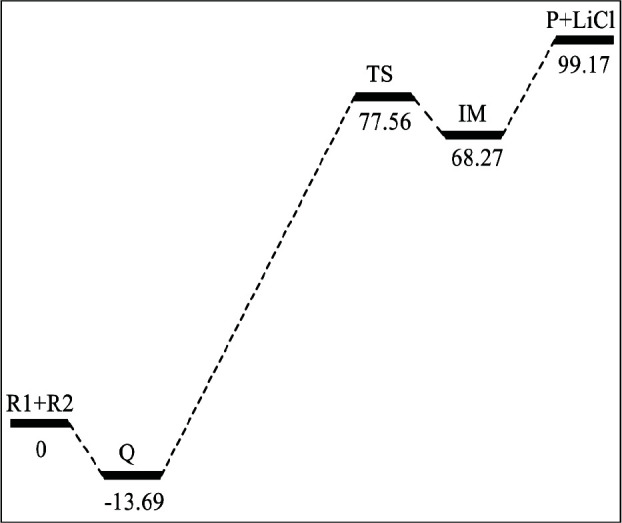
The potential energy surface profile of step 1 (the relative energies are given in kJ/mol).

Based on the computation results, we found that along the potential energy surface there is one precursor complex (Q), one transition state (TS), and one intermediate (IM). The geometries of the stationary points which calculated at the M06-2X/BSI level are shown in Figure 1, and their relative energies are shown in Figure 2. The calculated structure coordinates of reactants, intermediates, transition states and products were shown in the supporting information.

One precursor complex Q will be formed when R1 approaches R2 as shown in Figure 1. The distances of O-Ge and C-Ge are 0.267 and 0.355 nm respectively and the C-O-Ge angle is 129.4 degrees. These facts indicated the interaction between R1 and R2 is weak and the reaction will proceed further. As shown in Figure 2, the relative energy of Q is –13.69 kJ/mol.

Due to the rotation of acetone(<90º), the distance of O-Ge and C-Ge are becoming shorter and the C-O-Ge angle is becoming smaller, a transition state (TS) is formed. As shown in Figure 1, in TS the distance of O-Ge and C-Ge are 0.212 and 0.201 nm respectively, and the C-O-Ge angle is only 70.5 degrees. The IRC results show that TS is correctly in the middle of Q and intermediate IM. The imaginary frequency of TS is 207.0i cm^–1^. The relative energy of TS is 77.56 kJ/mol, consequently, the barrier height is 91.25 kJ/mol.

As reaction goes on, the intermediate IM will be formed after TS, and it can be regarded as the complex of the last products LiCl and *c* -H_2_GeOC(CH_3_)_2 _(P). As shown in Figure 1, in IM the distances of O-Ge and C-Ge of IM further decrease to 0.187 and 0.192 nm respectively, and the angle of C-O-Ge is 69.4 degrees, which indicates the formation of O-Ge and C-Ge bonds. As shown in Figure 2, the relative energy of IM is 68.27 kJ/mol.

The reaction of H_2_GeLiCl (R1) and CH_3_COCH_3 _(R2) resulted in the formation of LiCl and *c* -H_2_GeOC(CH_3_)_2 _(P) in the first step. The relative energies of the products (LiCl + *c* -H_2_GeOC(CH_3_)_2_) is 99.17 kJ/mol. It can be seen that the addition reaction of H_2_GeLiCl with acetone is endothermic in the first step.

### 3.2. Step 2 of the addition reaction of H2GeLiCl with acetone

**Figure 3:**
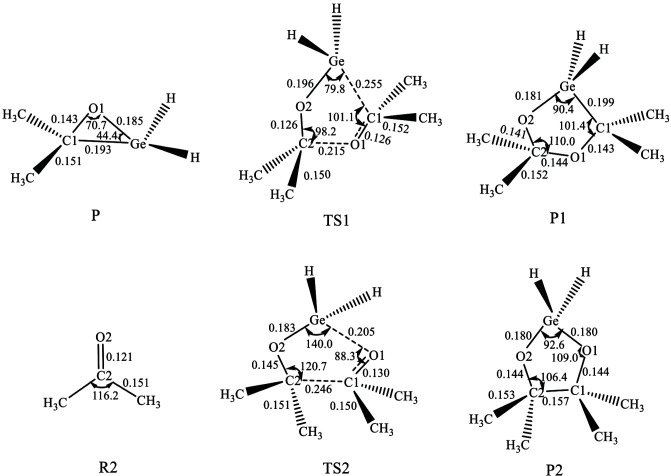
The geometries of the stationary points along the potential energy surfaces of the addition reaction of c-H_2_GeOC(CH_3_)_2_ and acetone optimized at the M06-2X/6-311+G (d,p) level (bond lengths are given in nm and angles in degrees).

**Figure 4:**
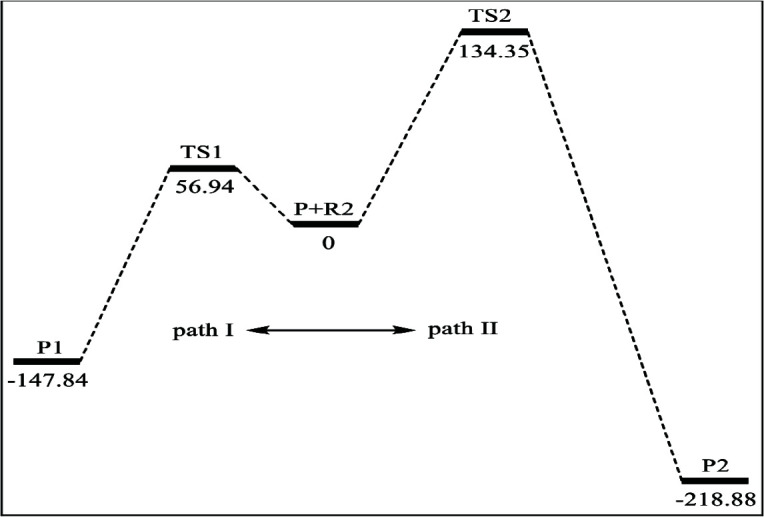
The potential energy surface profile of path I and path II (the relative energies are given in kJ/mol).

The M06-2X/BSI calculations indicate that there are two possible pathways (path I and II) in the reaction of *c* -H_2_GeOC(CH_3_)_2_ and CH_3_COCH_3_. The Ge atom of *c* -H_2_GeOC(CH_3_)_2_ approaches the O atom of CH_3_COCH_3_ in the path I and path II and then interacts with each other. But the interaction from different directions results in different transition states and ultimate products. In path I, the O1-Ge bond of *c* -H_2_GeOC(CH_3_)_2 _and the O2-C2 bond of CH_3_COCH_3_ are spatially close in a parallel manner, and then the O1-Ge bond of *c* -H_2_GeOC(CH_3_)_2_ is broken, with the Ge atom approaching and interacting with the O2 atom of CH_3_COCH_3_, at the same time, the O1 atom of *c* -H_2_GeOC(CH_3_)_2_ approaches and interacts with the C2 atom of CH_3_COCH_3_ to form the product 2,4-dioxagermolane (P1). However, in path II, the O2-C2 bond of CH_3_COCH_3_ is not parallel to the O1-Ge bond of *c* -H_2_GeOC(CH_3_)_2_ but approaching the C1-Ge bond of *c* -H_2_GeOC(CH_3_)_2 _in an almost parallel manner. After the C1-Ge bond of *c* -H_2_GeOC(CH_3_)_2 _is broken, the Ge atom approaches and interacts with the O2 atom of CH_3_COCH_3_ and the C1 atom of *c* -H_2_GeOC(CH_3_)_2_ approaches and interacts with the C2 atom of CH_3_COCH_3 _causing the formation the product 2,5-dioxagermolane (P2). As shown in Figure 3, TS1 is the transition state of path I, of which unique imaginary frequency is 231.82i cm^–1^ calculated by M06-2X/BSI. The relative energy of TS1 is 56.94 kJ/mol as depicted in Figure 4, which is the barrier height of path I. A spiro-Ge-heterocyclic product (P1), 2,4-dioxagermolane, is formed at the end of path I, and the relative energies of P1 is –147.84 kJ/mol.

In path II, TS2 is the transition state as shown in Figure 3. The unique imaginary frequency of TS2 is 406.6i cm^–1^ calculated by M06-2X/BSI. The relative energy of TS2 is 134.35 kJ/mol (in Figure 4), which is the barrier height of path II. At the end of path II, the other spiro-Ge-heterocyclic product (P2) forms, which is the 2,5-dioxagermolane. The relative energy of P2 is –218.88 kJ/mol.

We can find that path I and II are both exothermic. The barrier height of path I is about 77.41 kJ/mol lower than path II which can be concluded from Figure 4. Then we can draw a conclusion that the path I is more favorable dynamically.

### 3.3. The mechanism of addition reactions

For studying the addition reaction pathway, the IRC analysis was carried out on the basis of the TS, TS1, and TS2 to study the interactions of the step 1 and the step 2 respectively in the addition reaction.

**Figure 5:**
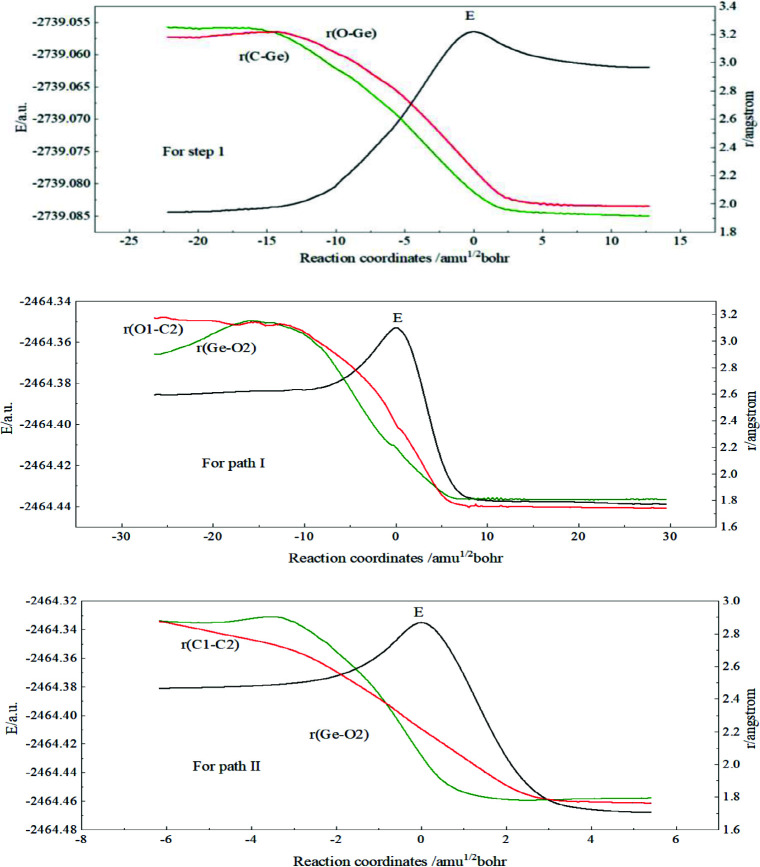
The calculated IRC results of three transition states.

We took the first step as an example. The total energy (E) of the reactants and the bond lengths of C-Ge and O-Ge are shown in Figure 5 along the reaction pathway. When the reaction coordinates rise from –5.0 to 0.0, the total energy (E) increases rapidly and reaches a peak. Also, the bond lengths of C-Ge and O-Ge gradually decrease to a stable value, which indicates the formation of C-Ge and O-Ge bonds. Consequently, TS connects Q and IM correctly. In the two paths of the step 2, the results of IRC calculations of the TS1 and TS2 are also shown in Figure 5 and both of them are correct.

## 4. Conclusion

By using the DFT M06-2X and QCISD methods, firstly we studied the addition reaction of the germylenoid H_2_GeLiCl with acetone. The geometry optimizations were carried out at M06-2X/6-311+G (d,p) level and the single-point energies were computed at the QCISD/6-311++G (d,p) level in sequence. The results reveal that there are two ways existing for the reaction of H_2_GeLiCl and CH_3_COCH_3_, and different products are formed: 2,4-dioxagermolane (P1) and 2,5-dioxagermolane(P2), respectively. The process can be viewed as a two-step reaction and the step 1is the same for two products. In step 1, H_2_GeLiCl and acetone react and oxagermacyclopropane (P) is formed by an addition reaction. There is a precursor complex, a transition state and an intermediate existing in step I along the potential energy surface. In step 2, there is a continuous reaction of product oxagermacyclopropane (P) with acetone, and the different bond-forming ways of them lead to the formation of different spiro-Ge-heterocyclic products. There are two possible pathways (I and II), which finally form spiro-Ge-heterocyclic products 2,4-dioxagermolane (P1) and 2,5-dioxagermolane (P2), respectively. The barrier height of path I is 56.94 kJ/mol while the barrier height of path II is 134.35 kJ/mol, which means the path I favors thermodynamically. Therefore, it can be concluded that the dominant channel of the addition reaction of H_2_GeLiCl and CH_3_COCH_3_ is that firstly R1 + R2 → P and then P + R2→ P1. We are confident that this work will provide a new inspiration for the synthesis of new germanium-containing compounds.

Supplementary Materials
