# Supplementary material for: Theoretical investigation on the addition reaction of the germylenoid H2GeLiCl with acetone
Source: Turk J Chem. 2021 Aug 27;45(4):1125–32. doi: 10.3906/kim-2012-35 (PMC8520399; doi:10.3906/kim-2012-35)
Supplement: Supplementary file 1 — Supplementary Materials [file turkjchem-45-1125-sup001.pdf]

Supporting information

The structure coordinates of reactants, intermediates, transition states, and products:

R1:

Ge	−2.56829	−0.28241	−0.00284
H	−2.78369	0.89251	1.12108
H	−2.78763	0.90659	−1.11107
Li	−4.23708	1.59101	0.01196
Cl	−5.0275	−0.44301	0.00049

R2:

C	2.50731	−0.37278	0.60534
O	1.54757	0.29175	0.30668
C	2.3953	−1.85413	0.89342
H	2.7478	−2.06328	1.90679
H	1.36199	−2.17702	0.78596
H	3.03472	−2.41561	0.20729
C	3.89238	0.22709	0.71334
H	4.28682	0.07265	1.72104
H	4.57088	−0.27759	0.02064
H	3.85582	1.29078	0.48805

Q:

Ge	−0.84749	−0.46504	0.0182
H	−0.75031	0.8763	0.93765
H	−0.79836	0.5103	−1.28457
Li	−1.94153	1.76091	−0.32943
C	2.70521	−0.43519	0.04558
O	1.71284	0.23225	−0.14528
C	4.08214	0.15143	−0.14277
H	4.73268	−0.11611	0.69264
H	4.52445	−0.27635	−1.0474
H	4.01855	1.23231	−0.24837
C	2.63324	−1.87709	0.47914
H	3.33185	−2.48565	−0.09904
H	2.93975	−1.94048	1.5276
H	1.61797	−2.25726	0.37951
Cl	−3.35742	0.15328	−0.03065

TS:

Ge	−0.28391	−0.30278	0.07138
H	−0.7351	−0.52581	−1.43903
H	−0.59787	−1.59492	0.83841
Li	−2.5419	−0.23429	−1.63119
C	1.72312	0.25602	−0.00533
O	1.64948	−0.96084	−0.4839
Cl	−3.02224	0.36362	0.30496
C	2.32919	0.436	1.37158
H	3.41852	0.38519	1.25455
H	2.06628	1.39989	1.80745
H	2.02546	−0.37135	2.03752
C	2.00111	1.38179	−0.97981
H	1.73052	2.35288	−0.56511
H	3.07819	1.37311	−1.18573
H	1.47894	1.21564	−1.92178

IM:

Ge	0.13164	0.34786	−0.13985
H	−0.55325	0.72195	1.22229
H	−0.09623	1.35683	−1.25292
Li	−2.2985	−0.04348	1.35567
C	1.43682	−1.05163	−0.05039
O	1.94242	0.2336	0.32457
Cl	−2.67869	−0.46143	−0.63336
C	2.04589	−1.59632	−1.32161
H	3.08475	−1.87996	−1.11644
H	1.50407	−2.47651	−1.67312
H	2.05476	−0.84549	−2.11279
C	1.40887	−2.04325	1.09046
H	0.83067	−2.93194	0.82853
H	2.43729	−2.34825	1.31623
H	0.99435	−1.59505	1.99576

$LiCl^-$

Li	−3.85526	−1.28196	0.15847
Cl	−2.20558	−1.69782	−0.93094

P:

Ge	−0.51681	0.05007	0.00228
H	−1.26589	0.3489	1.3034
H	−1.27687	0.33029	−1.29664
C	1.28974	−0.6225	−0.00097
O	1.17372	0.80248	−0.01028
C	1.90178	−1.16101	1.27092
H	1.81037	−2.24763	1.32258
H	2.9658	−0.89824	1.28733
H	1.43533	−0.72135	2.1537
C	1.89037	−1.1783	−1.2709
H	2.95423	−0.91604	−1.30032
H	1.79832	−2.26548	−1.30735
H	1.4161	−0.7503	−2.15526

TS1:

Ge	−0.70694	−1.33075	−0.08446
H	−0.95848	−2.29472	−1.29068
H	−0.29844	−2.34449	1.04574
C	1.76486	0.07342	−0.04701
O	1.07275	−0.76459	−0.67773
C	−0.95839	1.20527	0.07982
O	0.23587	1.5563	0.27399
C	2.69522	0.9483	−0.81943
H	2.38818	0.98949	−1.86239
H	2.69543	1.94723	−0.38482
H	3.70703	0.53478	−0.75059
C	1.96295	−0.06431	1.43051
H	2.69793	−0.86179	1.59181
H	2.33201	0.86467	1.85934
H	1.03346	−0.34857	1.92268
C	−1.53259	1.41457	−1.31337
H	−2.49153	0.9144	−1.45055
H	−1.67228	2.49507	−1.44514
H	−0.82468	1.07352	−2.06981
C	−1.9361	1.35947	1.23366

H	−2.15024	2.43081	1.33377
H	−2.87238	0.83015	1.05967
H	−1.4839	1.01718	2.16396

P1:

Ge	0.88674	−0.02142	0.11379
H	1.67374	−0.33239	1.40507
H	1.80997	0.0172	−1.11503
C	−1.65884	−0.73743	0.22796
O	−0.40761	−1.25096	−0.17448
C	−0.48907	1.41459	0.26209
O	−1.69302	0.6808	0.0054
C	−0.37237	2.44679	−0.85503
H	−1.24619	3.10508	−0.83179
H	0.52489	3.05891	−0.73279
H	−0.33828	1.96385	−1.83346
C	−0.5531	2.10935	1.62063
H	0.34474	2.71128	1.78609
H	−1.42227	2.77397	1.64214
H	−0.64569	1.4054	2.4482
C	−1.89654	−1.06138	1.70109
H	−2.81251	−0.58355	2.0526
H	−1.97907	−2.14237	1.82426
H	−1.06061	−0.71879	2.31495
C	−2.73591	−1.31956	−0.66498
H	−2.74947	−2.40536	−0.56324
H	−3.71158	−0.91613	−0.38933
H	−2.5169	−1.05726	−1.69975

TS2:

Ge	0.68396	−1.10731	0.02721
H	0.58035	−1.9134	−1.26697
H	1.05877	−1.71586	1.37684
C	0.72405	0.98365	−0.04446
O	1.92038	0.19944	−0.30405
C	−1.48764	−0.09404	0.051
O	−1.30995	−1.30662	0.47915
C	−1.93054	0.06797	−1.38294
H	−2.95593	−0.32148	−1.40058
H	−1.94745	1.10311	−1.71394
H	−1.33971	−0.54049	−2.06654
C	−2.05019	0.89951	1.02385
H	−1.58753	0.79366	2.00311
H	−1.98008	1.92612	0.66667
H	−3.11139	0.63703	1.12129
C	0.49547	1.93061	−1.19704
H	−0.37364	2.57572	−1.0429
H	1.38496	2.56806	−1.27395
H	0.39034	1.39974	−2.14432
C	0.88985	1.7115	1.27763
H	1.84541	2.24681	1.2292
H	0.09822	2.43887	1.46224
H	0.94201	1.02692	2.12797

P2:

Ge	−1.65214	0.00018	−0.00014
H	−2.49414	−0.55066	1.16046
H	−2.49416	0.5511	−1.16068

C	0.86894	0.78242	0.07591
O	–0.41121	1.13601	0.62786
C	0.86872	–0.78264	–0.07579
O	–0.41136	–1.1358	–0.62817
C	1.00967	–1.47691	1.2795
H	0.78297	–2.53656	1.15089
H	2.02269	–1.38313	1.67616
H	0.31468	–1.0573	2.01238
C	1.92721	–1.29035	–1.04384
H	2.92502	–0.96276	–0.74027
H	1.91077	–2.38167	–1.05491
H	1.72614	–0.93648	–2.0548
C	1.92728	1.28978	1.04431
H	2.92509	0.96189	0.74104
H	1.91117	2.3811	1.05539
H	1.72579	0.93595	2.0552
C	1.01054	1.47664	–1.27933
H	0.78416	2.53636	–1.1508
H	2.02365	1.38251	–1.67569
H	0.31563	1.05724	–2.01241
